# A novel 3-hydroxypropionic acid-inducible promoter regulated by the LysR-type transcriptional activator protein MmsR of *Pseudomonas denitrificans*

**DOI:** 10.1038/s41598-019-41785-y

**Published:** 2019-03-29

**Authors:** Nam Hoai Nguyen, Satish Kumar Ainala, Shengfang Zhou, Sunghoon Park

**Affiliations:** 1 School of Chemical and Biomolecular Engineering, Pusan National University, Busan, 46241 Republic of Korea; 2 School of Energy and Chemical Engineering, UNIST, UNIST-gil 50, Ulsan, 44919 Republic of Korea; 3 Key Laboratory of Biotechnology for Medicinal Plant, School of Life Sciences, Jiangsu Normal University, Xuzhou City, 221116 China

## Abstract

MmsR (33.3 kDa) is a putative LysR-type transcriptional activator of *Pseudomonas denitrificans*. With the help of 3-hydroxypropionic acid (3-HP), an important platform chemical, MmsR positively regulates the expression of *mmsA*, which encodes methylmalonylsemialdehyde dehydrogenase, the enzyme involved in valine degradation. In the present study, the cellular function of MmsR and its binding to the regulatory DNA sequence of *mmsA* expression were investigated both *in vivo* and *in vitro*. Transcription of the *mmsA* was enhanced >140-fold in the presence of 3-HP. In the MmsR-responsive promoter region, two operators showing dyad symmetry, designated O_1_ and O_2_ and centered at the −79 and −28 positions, respectively, were present upstream of the *mmsA* transcription start site. An electrophoretic mobility shift assay indicated that MmsR binds to both operator sites for transcription activation, probably in cooperative manner. When either O_1_ or O_2_ or both regions were mutated, the inducibility by the MmsR-3-HP complex was significantly reduced or completely removed, indicating that both sites are required for transcription activation. A 3-HP sensor was developed by connecting the activation of MmsR to a green fluorescent readout. A more than 50-fold induction by 25 mM 3-HP was observed.

## Introduction

3-Hydroxypropionic acid (3-HP) is a commercially important platform chemical that can be converted to acrylic acid, malonic acid, acrylamide, 1,3-propanediol and other valuable chemicals. Biologically, 3-HP can be produced from glucose or glycerol^1^. With glucose as the carbon source, 3-HP can be synthesized via malonyl-CoA or β-alanine. With glycerol as the carbon source, 3-HP is synthesized by two enzymatic reactions, catalyzed by the coenzyme B_12_-dependent glycerol dehydratase converting glycerol to 3-hydroxypropionaldehyde (3-HPA) and the 3-HPA-specific aldehyde dehydrogenase oxidizing 3-HPA to 3-HP, respectively. Several microorganisms, including *Escherichia coli*, *Klebsiella pneumoniae*, *Pseudomonas denitrificans* and *Saccharomyces cerevisiae*, have been developed as hosts^2,3^. Among them, *K*. *pneumoniae* and *P*. *denitrificans* are advantageous when glycerol is used as the carbon source because they can naturally synthesize coenzyme B_12_^4,5^.

**Figure 1:**
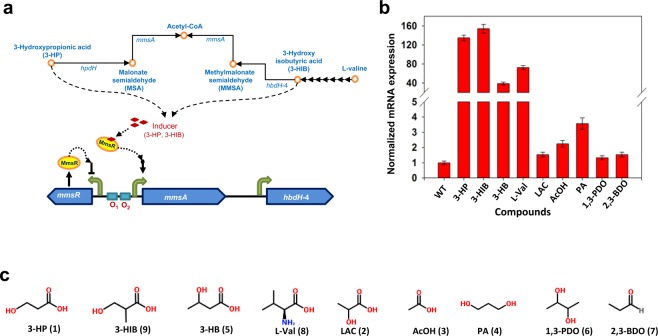
Regulation of gene expression by the LysR-type transcriptional activator protein, MmsR, in 3-hydroxypropionate degrading pathway of *Pseudomonas denitrificans*. (**a**) Hypothetical schematic representation of MmsR-based regulation. *hpdH*, 3-hydroxypropionate dehydrogenase; *mmsA*, methylmalonate semialdehyde dehydrogenase; *hbdH*4, 3-hydroxyisobutyrate dehydrogenase IV. (**b**) Relative mRNA abundance of *mmsA* in the presence of various candidate inducer molecules (25 mM). The amount of transcript was determined by real-time PCR analysis and ΔΔCT method as described previously^25^. The data were normalized against the un-induced mRNA expression level of *mmsA* in the wildtype strain, which was ~10-fold lower than that of the reference gene, *rpoD*. (**c**) Structures of chemical compounds tested for inducer specificity. 3-HP (**1**), 3-hydroxypropionic acid; 3-HIB (**9**), 3-hydroxyisobutyrate; 3-HB (**5**), 3-hydroxybutyrate; L-val (**8**), L-valine; LAC (**2**), L-lactate; AcOH (**3**), acetate; PA (**4**), propionaldehyde; 1,3-PDO (**6**), 1,3-propanediol; and 2,3-BDO (**7**), 2,3-butanediol.

In the course of the development of *P*. *denitrificans* as the 3-HP production host, we observed that 3-HP was actively degraded^6^. Several enzymes, namely putative 3-hydroxyisobutyrate dehydrogenase IV (HbdH-4), 3-hydroxypropionate dehydrogenase (HpdH) and/or methylmalonate semialdehyde dehydrogenase (MmsA), were involved in 3-HP degradation^7^ (see Fig. 1a for the gene arrangement of *mmsA* and *hbdH*4; *hpdH* is present in a separate operon). Furthermore, we found that the transcription of the *mmsA* gene encoding the MmsA enzyme was up-regulated at a high level (>100-fold) but only in the presence of 3-HP^8^. This suggests that there exists positive regulation on the expression of the enzyme, where 3-HP acts as an inducer. Analysis of gene arrangement near *mmsA* revealed the presence of a putative LysR-family transcriptional regulator protein that could be transcribed in the opposite direction of *mmsA*. It was speculated that the transcriptional regulator protein, when complexed with 3-HP, activates the transcription of *mmsA*. Considering the nature of this promoter (inducible by 3-HP) and its high induction efficiency (over 100-fold), the promoter should be useful for the expression of pathway enzymes for 3-HP synthesis or development of 3-HP-responsive biosensors. However, the biochemical characteristics of the transcriptional activator protein, including its binding sequences, have not yet been revealed.

This study aimed to elucidate the 3-HP-inducible gene regulation necessary for the expression of *mmsA* at the cellular and biochemical levels. At the cellular level, the specificity and/or spectrum of the small-molecule inducer(s) was studied using various acids structurally similar to 3-HP or the intermediates appearing in the L-valine degradation pathway and central carbon metabolism. The role of MmsR, the putative transcriptional activator protein, was confirmed by the removal of *mmsR* from the chromosome and subsequent complementation. Additionally, the promoter region that contains two operator sites (O_1_ and O_2_) was characterized by site-directed mutagenesis on each or both sites. At the biochemical level, the interactions of MmsR protein, 3-HP and the promoter region of *mmsA* were investigated by an electrophoretic mobility shift assay and DNase I footprinting using purified recombinant MmsR. Finally, a biosensor for the detection of 3-HP was developed and tested by expressing a green fluorescent protein (GFP) under the promoter activated by MmsR. This study should be useful for developing efficient 3-HP synthetic pathways and 3-HP sensors in many microorganisms.

## Results and Discussion

### Specificity of small-molecule inducers for MmsR transcriptional activator protein

According to our previous study, the putative transcription activator protein MmsR (previously designated C4-LysR) is composed of the N-terminal helix-turn-helix domain for DNA binding, the C-terminal domain for inducer binding that responds to 3-HP, and a linker connecting the two domains^8^. The spectrum of the inducer molecules for MmsR was studied using various acids and alcohols, such as L-lactic acid (LAC (**2**)), acetic acid (AcOH (**3**)), propionic acid (PA (**4**)), 3-hydroxybutyrate (3-HB (**5**)), 1,3-propanediol (1,3-PDO (**6**)) and 2,3-butanediol (2,3-BDO (**7**)), as well as L-valine (L-val (**8**)) and its degradation intermediate 3-hydroxyisobutyrate (3-HIB (**9**)). Most of the acids and alcohols tested were chosen mainly due to their similarity to 3-HP in size and/or structure. On the other hand, L-val and 3-HIB were chosen because *mmsA*, whose transcription was regulated by MmsR, encodes methylmalonylsemialdehyde dehydrogenase, an enzyme involved in L-val degradation. *P*. *denitrificans* was cultured on minimal medium with and without each compound to be tested, and transcription of *mmsA* was measured by quantitative RT-PCR (Fig. 1b). The housekeeping gene *rpoD*, encoding sigma factor 70, was used as a reference. Transcription of the *mmsA* gene was enhanced markedly upon exposure of *P*. *denitrificans* to chemicals compared to basal expression level in non-induction condition, such as 3-HIB (**9**) (154-fold), 3-HB (**5**) (38-fold) and L-val (**8**) (72-fold), in addition to 3-HP (**1**) (134-fold). By contrast, limited or no induction was observed after exposure to LAC (**2**), AcOH (**3**), PA (**4**), 1,3-PDO (**6**) and 2,3-BDO (**7**). As with all β-hydroxy acids, the chemicals 3-HP, 3-HB and 3-HIB are structurally similar (Fig. 1c), and the structural feature of having both the carboxyl and β-hydroxy groups seems to be integral to the ability to bind to MmsR. L-val, structurally much different from these three compounds, is converted to 3-HIB, and the induction by L-val is attributable to L-val-derived 3-HIB rather than to L-val itself (Fig. 1a).

**Figure 2:**
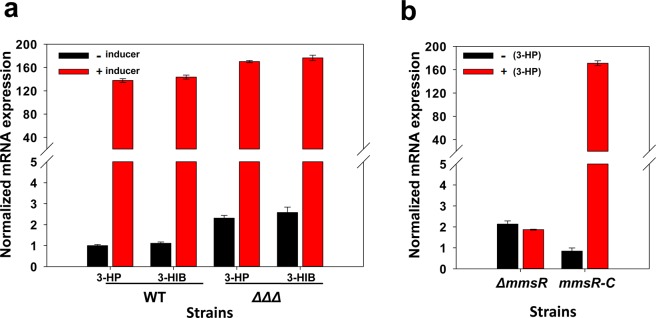
Transcriptional analysis of *mmsA* in *P*. *denitrificans* with various genetic backgrounds. (**a**) Transcription of *mmsA* in the wildtype (WT) and mutant *P*. *denitrificans*Δ*hpdH*Δ*hbdH4*Δ*hbdH1* (ΔΔΔ) strains. 3-HP and 3-HIB (each at 25 mM) were used as inducers. (**b**) Deletion and complementation of transcriptional activator, MmsR. Δ*mmsR*, *P*. *denitrificans*Δ*mmsR*; and *mmsR*-C, *P*. *denitrificans*Δ*mmsR* but with *mmsR*-complementation from a plasmid. The data were normalized against the un-induced mRNA expression level of *mmsA* in the wildtype strain, which was ~10-fold lower than that of the reference gene, *rpoD*.

We also examined the possibility that malonate semialdehyde (MSA, derived from 3-HP) and methylmalonate semialdehyde (MMSA, derived from L-val and 3-HIB) rather than 3-HP and/or 3-HIB are the physiological inducers (see Figs 1a and 2a). In general, aldehydes are toxic, and aldehyde-degrading genes are often up-regulated when aldehydes are accumulated^9^. According to our previous study^6^, 3-HP was degraded by the enzymes encoded by *hpdH*, *hbdH*4 and *hbdH*1, and the deletion mutant *P*. *denitrificans* Δ*hpdH*Δ*hbdH*4Δ*hbdH*1 did not degrade 3-HP at an appreciable rate. As shown in Fig. 2a, in the triple mutant, which should not produce MSA from 3-HP, transcription of *mmsA* was nonetheless up-regulated by 3-HP. This indicates that 3-HP can activate MmsR protein without being converted to MSA. Similarly, transcription of *mmsA* was up-regulated by 3-HIB in the triple mutant, suggesting that 3-HIB is also a real inducer of MmsR.

To confirm the physiological role of MmsR as a transcriptional activator, deletion and subsequent complementation experiments were conducted (Fig. 2b). In the deletion mutant (Δ*mmsR*), transcription of *mmsA* was low, and the level was not affected by 3-HP. On the other hand, when *mmsR* was re-introduced to the Δ*mmsR* mutant by a plasmid, up-regulation of *mmsA* by 3-HP was fully restored. These results confirm that MmsR is a real transcriptional activator protein for the expression of *mmsA*.

### *In vivo* characterization of *mmsA* promoter region

The transcriptional start site (TSS) of *mmsA* was identified by 5′ rapid amplification of cDNA ends (RACE)^10^. It was located at the G residue, −15 relative to the translation start of the *mmsA* structural gene (Supplementary Fig. S1). The putative −35 box (TGTTAG) and −10 box (AAAAAG) were predicted based on the TSS, which were spaced by 17 bp.

**Figure 3:**
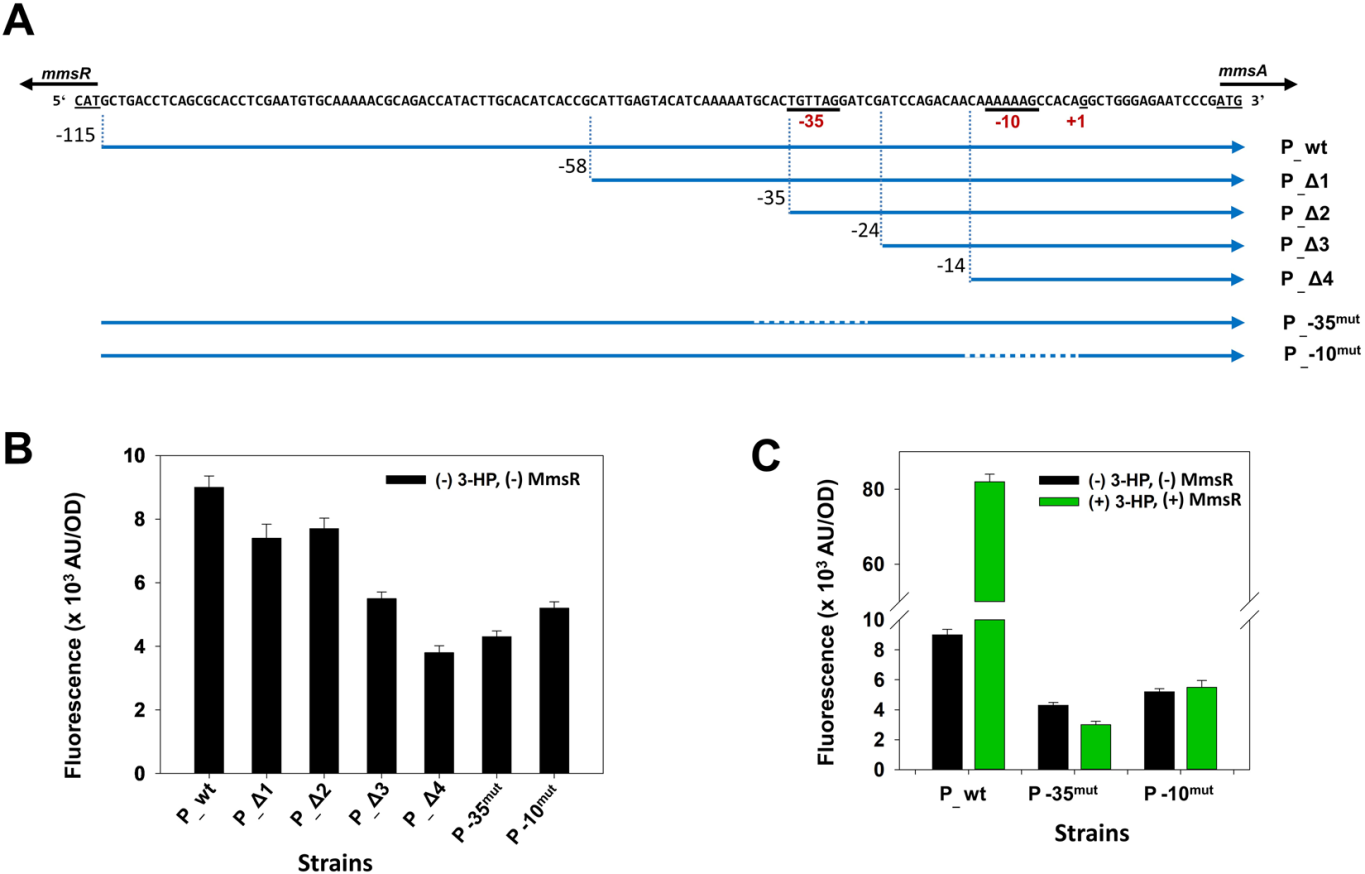
(**A**) Analysis of the *mmsR*-*mmsA* intergenic region for identification of the P_*mmsA*_ promoter. A series of deletion (P_Δ1 − P_Δ4) and site-directed mutagenesis (P-35^mut^ and P-10^mut^) are shown. Promoter elements (−10, −35) and TSS (+1) of P_*mmsA*_ promoter were underlined. Change in gene expression of basal level (**B**) and inducibility (**C**) of the *mmsA* promoter after deletion and/or site-directed mutagenesis. GFP intensity (AU/OD_600_; AU, arbitrary unit) was measured in the absence (−) of 3-HP and MmsR (black bar) or presence (+) of 3-HP and MmsR (green bar). 3-HP was supplemented at 25 mM.

Importance of −10 and −35 boxes was studied by 5′ end mapping and site-directed mutagenesis (Fig. 3). For the mapping experiment, the P_*mmsA*_ promoter region including the putative −10 and −35 regions were sequentially deleted and their strength was determined using GFP as reporter. *P*. *denitrificans* either lacking or expressing the *mmsR* gene was used as a host because MmsR considerably affects the promoter strength. In the Δ*mmsR* strain (un-induced condition), when the promoter region between −115 and −58 (P_Δ1) or −115 and −35 (P_Δ2) was deleted, the *gfp* expression decreased by approximately 16% compared to that of the control (P_wt), which contains the whole-length promoter. In comparison, when a longer upstream sequence before −24 (P_Δ3) or −14 (P_Δ4) was deleted, the promoter strength decreased more considerably by 39 or 58%, respectively (see Fig. 3B). These results suggest that the sequences deleted, from −35 to −14 relative to the TSS, in P_Δ3 and P_Δ4, should contain important elements for transcription, most likely the −10 or −35 regions. In addition, site-directed mutagenesis in the putative −10 (P-10^mut^) and −35 (P-35^mut^) regions reduced the promoter strength by 52% in comparison to that of P_wt (see Fig. 3B). The site-directed mutagenesis experiments have been repeated for the *mmsR*^+^ strain. Under the induced conditions with 3-HP, GFP intensity was high at 82 × 10^3^ AU/OD_600_. In comparison, GFP intensity after mutation of either the putative −10 box (P-10^mut^) or −35 (P-35^mut^) box was only 3–5 × 10^3^ AU/OD_600_ which is ~5% of that by P_wt (see Fig. 3B,C). When either the putative −10 or −35 box was deleted, presence of MmsR and 3-HP did not affect the *gfp* expression level (see Fig. 3C). Altogether, these results reveal the importance of the −10 and −35 regions which positioned from −34 to −29 and from −11 to −6 relative to the TSS, respectively, in P_*mmsA*_.

### *In vivo* characterization of *mmsA* operator region

**Figure 4:**
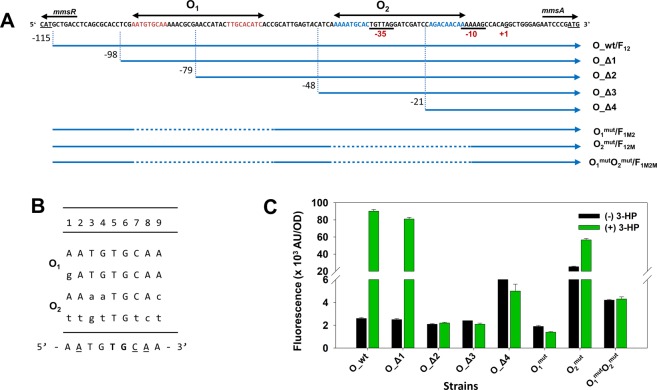
(**A**) Analysis of MmsR binding sites in the *mmsR*-*mmsA* intergenic region. A series of deletion (O_Δ1 – O_Δ4) and site-directed mutagenesis (O_1_^mut^, O_2_^mut^ and O_1_^mut^O_2_^mut^) are shown. The symbols O_1_ and O_2_ denote the probable tandem operator sequences corresponding to palindromic (red letters) and pseudo-palindromic (blue letters) regions recognized by the MmsR protein. Promoter elements (−10, −35) and TSS (+1) of P_*mmsA*_ promoter were underlined. F_12_, F_12M_, F_1M2_ and F_1M2M_ are DNA fragments used for *in vitro* electromobility shift assay (EMSA). F_12_ includes the complete intergenic region, where both O_1_ and O_2_ are intact; F_12M_ contains the intact O_1_ and mutant O_2_; F_1M2_ contains mutant O_1_ and intact O_2_; F_1M2M_ contains mutant O_1_ and mutant O_2_. (**B**) Consensus sequence between O_1_ and O_2_ operator sequences of *mmsR*-*mmsA* intergenic region. The 9 bp of each inverted half-site is shown in 5′ to 3′ direction. The lower letters indicate the mismatch of repeat with the consensus sequence. The residues in boldface in consensus sequence are converted in each repeat sequence. The residues in under lined in consensus sequence are highly conserved in three fragments. **(C)** Change in gene expression after deletion and/or site-directed mutagenesis of the *mmsA* operator region. GFP intensity (AU/OD_600_; AU, arbitrary unit) was measured in the absence (black bar) and presence (green bar) of 25 mM 3-HP. MmsR was constitutively expressed from plasmid under the weak promoter, P_*c1*_.

Before experimental characterization, *in silico* analysis of the operator region was briefly conducted^11^. Analysis by mfold web server^12^ identified the presence of a good stem-loop structure formed from dyad symmetry, often appearing as *cis*-acting element in many prokaryotic operators, with a spacer of 15 bp (see Supplementary Fig. S2a). This palindromic region was hypothesized as O_1_ site (from −95 to −63 relative to the TSS) and used as query for searching the second operator site O_2_, which is known to be less stringent, pseudo-palindrome. Two best candidates with good matches and spaced by 15 bp, AAAAATGCA-N_15_-CAGACAACAA (candidate 1, locating from −44 to −12 relative to the TSS) and TAGGATCGA-N_15_-AGCCACAGG (candidate 2, locating from −31 to +2 relative to the TSS) were identified (see Supplementary Fig. S2b). Furthermore, based on the typical location of O_2_ site (ABS) in relation to the promoter region in other LTTR systems^13–17^ (see Supplementary Fig. S2c), the first candidate, which was overlapped with the −35 element only, was suggested as the better candidate than the other one. The analysis shows that the dyad in the putative O_1_ site (from −95 to −63 relative to the TSS) is highly symmetrical, with only a single mismatch, while the inverted repeat in the putative O_2_ site (from −44 to −12 relative to the TSS) has 7 bases mismatched among the 9 (Fig. 4A). Alignment of the four palindromic fragments of the putative O_1_ and O_2_ operators showed the existence of the consensus sequence AATG**TG**CAA (Fig. 4B). Two bases T and G at positions 5 and 6 (bold), respectively, were fully conserved in all the fragments, and the three bases A, C, A at positions 2, 7 and 8 (underlined), respectively, were highly conserved in three fragments.

The 5′ end mapping and site-directed mutagenesis experiments were performed using GFP as reporter (Fig. 4A,C). To account for the possibility that *mmsR* is auto-regulated by itself and the auto-regulation interferes with the *gfp* transcription, *mmsR* was constitutively expressed from a plasmid under the control of the weak P_*c1*_ promoter (3*hibdH*-I promoter^18^) (see Materials and Methods). The sequences of O_wt (intact intergenic region) and O_Δ1 (downstream from position −98 relative to the TSS) that contain both the putative operators (O_1_ and O_2_) exhibited high 3-HP induction up to ~34.6-fold. In comparison, when removing sequence upstream from position −79 containing the first half site of the putative O_1_ site (O_Δ2) or further eliminating additional sequences down to −48 (O_Δ3) and −21 (O_Δ4) containing the other half site of O_1_ and O_2_ regions, respectively; inducibility was completely lost (see Fig. 4C). It is noticed that the basal level under MmsR (over)expression (O_Δ series) has been significantly decreased compared to that lacking MmsR (P_Δ series shown in Fig. 3) even though their sequence features are nearly identical. This suggests that the inducer-free MmsR protein represses the *mmsA* expression. Once the complete O_1_ site and half site of O_2_ are removed, this repression effect is abolished, as depicted by similar expression levels for O_Δ4 and P_Δ3. Effect of mutation (site-directed mutagenesis) in the O_1_ and/or O_2_ region on the promoter strength was also investigated. Here, the operator sequences were carefully modified to destroy the dyad symmetry without affecting the −35 region. The palindromic region of O_1_ site (AATGTGCAA-N_15_-TTGCACATC) was changed to ATCCTTGCT-N_15_-CGCCCAGGC in O_1_^mut^ or O_1_^mut^O_2_^mut^ mutations; while the sequence ATCCTTGCT-N_15_-CAGGCACAA was used to replace O_2_ site region (AAAATGCAC-N_15_-AGACAACAA) in O_2_^mut^ or O_1_^mut^O_2_^mut^ mutations, with the underlined letters indicated the modified nucleotides (see Supplementary Table S2 for whole sequences). As expected, mutation of O_1_ (O_1_^mut^) or both O_1_ and O_2_ (O_1_^mut^O_2_^mut^) completely abolished the inducibility by 3-HP. When the putative O_2_ region alone was mutated (O_2_^mut^), promoter strength in the absence of 3-HP greatly increased (~10-fold) compared to that of wild-type O_wt. When 3-HP is present, the strength was reduced by 37% (compared to that of wild type O_wt) but still remained at a relatively high level. This suggests that the O_2_ site has an important role in regulating P_*mmsA*_ function and strength, although its role or association with the O_1_ site is yet to be further elucidated. Collectively, the *in vivo* studies on the operator regions can be summarized as follows: (i) O_1_ alone can activate the transcription (with the help of 3-HP-MmsR complex), although less efficiently than when both O_1_ and O_2_ are present; (ii) binding of 3-HP-free MmsR to the O_2_ operator can suppress transcription from the P_*mmsA*_ promoter; and (iii) the high strength of the promoter of the O_2_ mutant still requires the presence of the O_1_ site. It is most likely that the P_*mmsA*_ promoter is regulated positively (in the presence of 3-HP) as well as negatively (in the absence of 3-HP).

The LTTR proteins and their *cis*-acting elements have been studied in several microorganisms. In the two symmetrical operator regions, termed RBS (Regulatory Binding Site) and ABS (Activator Binding Site), RBS was reported to have a better symmetry than ABS. In addition, the full sites of RBS and ABS were known to be often embraced by T-N_11_-A motifs. In *E*. *coli* and *Salmonella typhimurium* LT2, the IlvY protein (a LysR-type transcription regulatory protein) controlled the expression of *ilvC* gene (encoding acetohydroxy acid isomerase; EC 1.1.1.86)^19^. Transcription of the *ilvC* gene was induced by the substrates of acetohydroxy acid isomerase, acetohydroxybutyrate or acetolactate, and the induction was mediated by the IlvY protein. Similar to the P_*mmsA*_ promoter under study, the promoter region of the *ilvY* and *ilvC* genes had two operators, O_1_ and O_2_, and each operator was composed of 9-bp-long inverted repeats. The repeats shared homologous sequences, A(T)CATTGCAA in *E*. *coli* and AT(A)GTTGC(A)GN in *S*. *typhimurium* LT2, respectively. Moreover, similar to the case in *P*. *denitrificans*, symmetry between the two dyads in these strains was more stringent in O_1_ (a single mismatch) than in O_2_ (six mismatches). Another LysR-type activator AtzR, which is present in *Pseudomonas sp*., has been reported to show the dissimilarity in the ABS operator region. AtzR activates the expression of divergently transcribed cyanuric acid degradative operon *atzDEF*. However, ABS contained three motifs, designated ABS-1, ABS-2 and ABS-3, in which individual subsites had distinct roles in the activation process. *In vivo* mutational analysis showed that ABS-1 and ABS-2 subsites were required for full activation of the P_*aztDEF*_ promoter. In contrast, ABS-3 functioned as a ‘subunit trap’ leading to inactivation of P_*aztDEF*_ when AtzR was shifted to the ABS-2 and ABS-3 subsites^20^. Similar dual functions of the ABS operator region have been reported with CbbR^17^ and DntR^21^. With the P_*mmsA*_ under study, O_2_ site also seems to have the dual functions for both activation and repression. However, neither the presence of such multiple ABS’s nor shift of MmsR protein has been identified. For other LysR proteins such as IlvY^14^, CatR^16^, ClcR^15^ and HpaR^22^, the dual functions of O_2_ site has also been reported but without multiple ABS shown in AtzR. Further investigations are required to clarify the location of the O_2_ site(s) in the P_*mmsA*_ promoter and the mechanism for the dual functions, if present.

### Production of MmsR protein and its binding to operator sites *in vitro*

For biochemical characterization *in vitro*, a recombinant MmsR protein tagged with six histidine residues at the C-terminus of the protein was produced and purified from recombinant *E*. *coli*. The His-tag MmsR protein at either the C- or N-terminus showed the same performance as the native MmsR protein in the complementation experiments described in Fig. 2b (see Supplementary Fig. S3 for details); thus, one recombinant MmsR, C-His-tagged only, was studied *in vitro*. After careful optimization of the culture conditions (temperature, culture medium, IPTG concentration, harvest time and 3-HP concentration as well as co-expression with various chaperones, such as GroEL-ES, DnaKJ-GrpE and trigger factors; see Supplementary Fig. S4), the recombinant MmsR could be expressed in soluble form at a high level in *E*. *coli* by co-expression of *mmsR*-expressing plasmid and pGro7 containing groES-groEL chaperones (see Supplementary Fig. S4b) and purified by affinity chromatography (see Supplementary Fig. S5a). The size of the His-tag-fused MmsR protein was estimated to be 34.4 kDa, which is in good agreement with the size estimate based on the 6x *his*-*mmsR* gene sequence. According to native PAGE and/or gel filtration analyses, the MmsR protein was monomeric at the low concentration of 65 nM, while dimeric at the high concentration of 550 nM (see Supplementary Figs S5b and S7).

**Figure 5:**
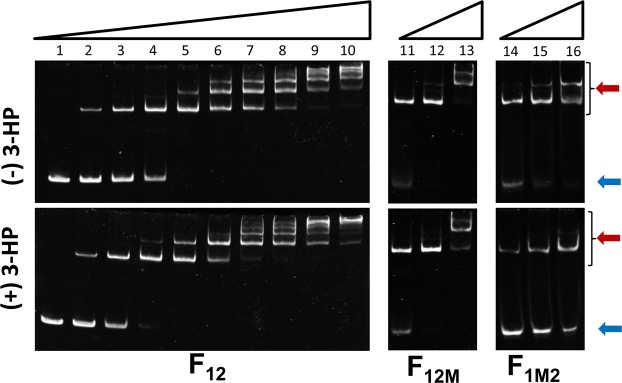
*In vitro* electromobility shift assay (EMSA) to study binding of the MmsR protein to operator sites. Three DNA fragments containing intact O_1_and O_2_ (F_12_), or mutated in either O_2_ site (F_12M_) or O_1_ site (F_1M2_) were used as probe. The experiments were conducted in the absence (upper panel) or presence (lower panel) of 3-HP (at 25 mM). Lanes 1–10, increasing amounts of the MmsR protein at 0, 0.36, 0.73, 1.45, 2.9, 5.8, 11.6, 14.5, 24.2 and 48.5 nM. Lane 11–13 and 14–15, increasing amounts of the MmsR protein at 2.9, 11.6 and 48.5 nM. The red arrows indicate the shifted complexes. The concentration of DNA fragments was fixed at 40 nM. Figures for EMSA experiments of F_12M_ and F_1M2_ were cropped from full-length gels shown in Supplementary Fig. S6.

The binding ability of MmsR to the *mmsR*-*mmsA* intergenic region was studied *in vitro* by EMSA (Fig. 5 and Supplementary Fig. S6). A total of 40 nM of the complete 130 bp DNA fragment containing the sequence from −115 to +16 relative to the TSS was used as a probe (designated the F_12_ fragment; see Fig. 4A), and the purified MmsR protein at varying concentration at 0–48.5 nM was incubated with the DNA probe in both the presence and absence of 3-HP as an inducer. Similar to *in vivo* experiment, the sequences, ATCCTTGCT-N_15_-CGCCCAGGC and ATCCTTGCT-N_15_-CAGGCACAA, were applied to destroy the dyad symmetry in O_1_ and O_2_ sites, respectively, for generation of F_1M2_, F_12M_, and F_1M2M_ fragments (see Fig. 4A and Supplementary Table S2 for sequence information). The DNA fragment mutated in both O_1_ and O_2_ (F_1M2M_) was used as a negative control. In electrophoresis, band shift appeared for the intact 130 bp DNA fragment when MmsR was added, and the ratio of the shifted DNA to un-shifted DNA increased upon amplifying the concentration of the MmsR protein during incubation. In contrast, no such mobility retardation was observed with the control DNA fragment up to 72.7 nM of MmsR protein (data not shown). This indicates that MmsR has a strong binding affinity to the native P_*mmsA*_, and they form a binding complex *in vitro*. At high concentrations of the MmsR protein, multiple bands traveling shorter distances appeared (see Fig. 5). It may attribute to the formation of diverse oligomeric complexes among the DNA fragments and protein molecules. The DNA probe has two binding sites (O_1_ and O_2_) for MmsR, and MmsR binds to the DNA as a dimer; thus, it is highly probable that DNA and protein form diverse oligomeric complexes when their concentrations are high. Figure 5 also shows the effect of 3-HP on binding between MmsR and the P_*mmsA*_ promoter. In the presence of 3-HP, intensity of the shifted band increased at lower MmsR concentrations (for a clear comparison, see lane 4 in the panels of F_12_). This suggests that 3-HP promotes the binding affinity between MmsR and the P_*mmsA*_ promoter. The EMSA experiments were also conducted with two DNA fragments having mutation (site-directed mutagenesis) in the palindromic regions of either O_1_ (F_1M2_) or O_2_ (F_12M_) operator site (see Fig. 4A and Supplementary Table S2 for sequence information). Under the same incubation condition, more intensive shifted-bands were observed with F_12M_compared to that with F_1M2_ in either absence or presence of 3-HP. This suggests higher affinity of MmsR toward O_1_ operator than O_2_ operator. Interestingly, the addition of 3-HP greatly reduced the binding of MmsR to the O_2_ region (see the panels for F_1M2_ in Fig. 5). This suggests that, along with the DNase I footprinting results (see below), ABS region should become less occupied by MmsR in the presence of 3-HP. Rhee *et al*. also studied the binding affinity of *E*. *coli* IlvY protein to DNA fragments containing either the tandem operators (O_1_O_2_) or the O_1_ or O_2_ operator alone. The binding affinity was highest with the fragment containing the tandem O_1_O_2_ operator and lowest with the O_2_ operator. However, in their system, the inducer molecule did not affect the binding of IlvY protein to the operators^14^.

### DNAase I footprinting on operator-promoter region

**Figure 6:**
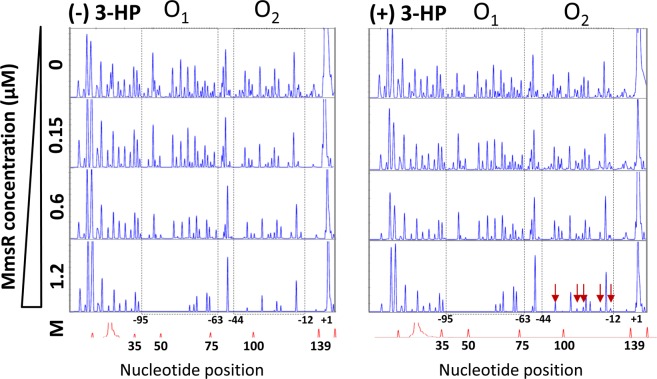
DNase I footprinting experiments on the *mmsR*-*mmsA* intergenic region. FAM-labeled *mmsR*-*mmsA* intergenic region was digested by DNase I in the absence (left) and presence (right) of 3-HP and analyzed using capillary electrophoresis. Sequence positions (x-axis) are marked with reference to the ROX 500 internal size-standard (M). The y-axis indicates relative fluorescent units (ABI 3130). MmsR concentration increased from 0 to 1.2 µM (concentrations were indicated on the left hand side). Dotted two black boxes (putative O_1_ and O_2_) in both panels indicate that DNase I digestion is inhibited by MmsR in dose-dependent manner. Red arrows highlight the change in protection by added 3-HP.

The MmsR binding regions was also studied by DNase I footprinting based on capillary electrophoresis (Fig. 6). The longer 169 bp DNA fragment that includes the complete 130 bp intergenic region (employed in the previous EMSA experiments) was used as a probe (Supplementary Table S2). DNA concentration was fixed at 230 nM, while MmsR concentration varied in 0–1.2 μM. The footprinting results exhibit the presence of two protected regions by MmsR, which should correspond to operator O_1_ (centered at position −79) and O_2_ (centered at position −28), respectively. Protection became more evident as MmsR concentration increased. However, it was difficult to pinpoint the boundary of protection at the base-pair level of the DNA sequence. Between the O_1_ and O_2_ operators, the former was more protected than the latter and this confirms the higher affinity of the O_1_ site than the O_2_ site toward MmsR. Addition of 3-HP barely affected the DNase I footprinting for the O_1_ operator. However, for the O_2_ operator, as indicated by appearance of more unprotected peaks (at the high MmsR concentration of 1.2 μM), a weaker protection was observed when 3-HP was added (Fig. 6). This result agrees with EMSA experiment, i.e., in the presence of 3-HP, the O_2_ region is less susceptible to MmsR.

A relaxation of DNA bending in O_2_ or ABS region, as studied with AtzR^13^, IlvY^19^ and CatR^15^, was suggested as a common mechanism for transcription activation of LysR-type regulator. Two major mechanisms of LTTR-mediated transcription activation have been suggested; (i) ‘sliding dimer mechanism’ where a LysR protein, when bound with a inducer molecule, shifts from one ABS (called ‘downstream ABS’) to another ABS (‘upstream ABS’) leading to free −35 region, as studied with AtzR^13^, CbbR^17^ and DntR^23^, and (ii) ‘different-side binding model’ where RNAP and LysR can contact different sides of DNA helix, as studies with IlvY^19^, CatR^15^, and ClcR^15^. According to our study on the *mmsR*-*mmsA* intergenic region, no extra ABS is present and the P_*mmsA*_ promoter functions better when the −35 box was not or less occupied by MmsR. This eliminate the first mechanism, although does not provide evidences to support the second mechanism.

### Use of 3-HP-inducible promoter as 3-HP biosensor

**Figure 7:**
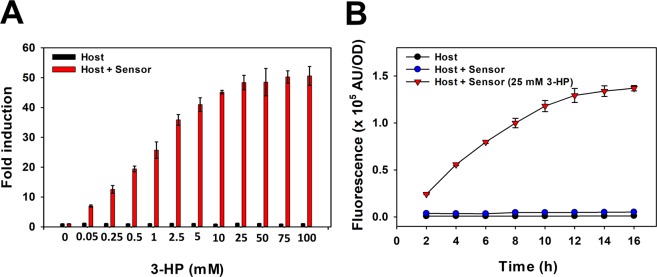
(**A**) The 3-HP triggered fluorescence in cells containing the biosensor (green bar) in a dose-dependent manner up to 25 mM of 3-HP when measured at 12 h. The host cells without the biosensor (black bar) did not respond to 3-HP. (**B**) The response of the biosensor to 25 mM 3-HP (red downward triangle) started immediately and continued to increase up to 12 h after induction. Basal induction in the host cells (black circle) or in the biosensor without 3-HP (blue circle) was low. Three independent measurements for each were conducted and the results were averaged.

The current dynamic promoter responding to 3-HP could be developed as a 3-HP sensor. The performance as a biosensor was briefly examined using the regulatory region containing both O_1_ and O_2_ sites (F_12_ fragment) with GFP as the reporter. MmsR protein was produced by the constitutive and weak promoters P_*c1*_ of *P*. *denitrificans* and from the same plasmid as GFP. When measured at 12 h, the fluorescent signal was enhanced as the 3-HP concentration increased to 25 mM, and the fold difference compared with the control (for which no 3-HP was added) was >50 (Fig. 7A). The derived time-course profile (Fig. 7B) shows the fluorescent signal increasing with time and 3-HP concentration. The response of the biosensor to 25 mM 3-HP started immediately and did not reach the point of saturation until after 12 h. It was noted that the background fluorescence of the control (without 3-HP) was low and was not different from that of the host *P*. *denitrificans* without the recombinant plasmid. This indicates that the transcription of *gfp* is blocked almost completely and that leaky expression is negligible when 3-HP is absent. We attributed this tight control of gene expression to the repressive function of 3-HP-free MmsR protein when bound to the O_2_ operator.

Recently, Rogers and Church reported development of 3-HP biosensors based on helper enzymes that convert 3-HP to the *acuR*-binding compound acrylate or *prpR*-binding 2-methylcitrate^24^. Both sensors could detect 3-HP successfully but only after conversion to acrylate or 2-methylcitrate. In comparison, the current MmsR biosensor directly recognizes 3-HP and does not require any helper enzymes. Furthermore, it generally has a better sensitivity and dynamic range. On this basis, we believe that the current biosensor is more convenient to use.

## Conclusions

MmsR in *P*. *denitrificans* functions as a transcriptional regulator activating the expression of the *mmsA* gene from its responsive promoter P_*mmsA*_. The fold increase in the transcription of *mmsA* by 3-HP induction was >140. The responsive promoter P_*mmsA*_ had two operator regions, designated O_1_ and O_2_, positioned at –79 and –28, respectively, upstream of the transcription start site of the *mmsA* gene. Binding of MmsR to O_1_ and O_2_ did not require an inducer molecule, but transcription activation strictly required it. Each of the O_1_ and O_2_ operators was composed of two 9-bp-long inverted repeats, and a mutation in the inverted repeats resulted in the reduction or complete loss of their binding capability to MmsR. The inducible promoter was developed as a biosensor for detection of 3-HP. With GFP as the reporter, the sensor showed a good response up to 25 mM 3-HP. This study will prove useful for the biotechnological application of the P_*mmsA*_ promoter.

## Materials and Methods

### Materials

*P*. *denitrificans* ATCC 13867 was purchased from ATCC (America). The primers were synthesized by Macrogen Co. Ltd (Seoul, Korea). 3-HP was purchased from Tokyo Kasei Kogyo Co. Ltd. (Tokyo, Japan) and TCI America (Portland). Unless indicated otherwise, all other chemicals and enzymes were purchased from Sigma-Aldrich (St. Louis, MO).

### Development of *P. denitrificansΔmmsR*

To construct the deletion mutant *P*. *denitrificans*Δ*mmsR*, the previously developed *sacB*-based method was used^5^. The pQSAK plasmid, which has a *sacB*-*Km* cassette, was used to knock off the *mmsR* gene from the chromosome (Supplementary Table S1). An engineered fragment containing the ~600–700 bp upstream (*QS*-*mmsR*-*US*) and downstream (*QS*-*mmsR*-*DS*) regions of the *mmsR* gene was generated by PCR from *P*. *denitrificans* genomic DNA. The fragment was cloned into the pQSAK vector using the GeneArt seamless cloning and assembly kit (Invitrogen, USA) and confirmed for sequence. The recombinant plasmid subsequently was used for homologous recombination.

### Complementation of *ΔmmsR* mutation and effect of His-tag on MmsR function

The ORF of the *mmsR* gene was PCR-amplified from *P*. *denitrificans* genomic DNA and overlapped with a native constitutive P_*c1*_ promoter (controlling expression of the *3hibdh*-I gene^18^) of *P*. *denitrificans*. The overlapped fragment was then cloned into the shuttle vector pUCPK’, which had been modified from the previously developed plasmid pUCPK’^5^. The resultant plasmid was designated pUCPK’/P_*c1*_-*mmsR*, and the recombinant plasmid was introduced by electroporation into the *P*. *denitrificans*Δ*mmsR* mutant to develop the complementation strain, *mmsR*-*C*. In order to evaluate the effect of His-tag on MmsR function, plasmids overexpressing the His-tagged MmsR at the N- or C-terminus were constructed. The resultant plasmids and recombinant strains were designated pUCPK’/P_*c1*_-*N6xhis*-*mmsR*, pUCPK’/P_*c1*_-*mmsR*-*C6xhis* and *N*-*his*-*mmsR*-*C*, *C*-*his*-*mmsR*-*C*.Details of primers were given in Supplementary Table S3.

### RNA extraction and Real-time PCR

The *P*. *denitrificans* strains were grown in M9-minimal medium containing 5 g/L sodium gluconate. The cells were cultivated under the aerobic condition at 37 °C and 200 rpm in an orbital incubator shaker. 3-HP and/or various chemicals (3-hydroxyisobutyrate, 3-hydroxybutyrate, L-valine, lactic acid, acetic acid, propionic acid, 1,3-propanediol and 2,3-butanediol) at 25 mM was supplemented at the OD_600_ of ~0.4–0.5. After a further 2 h cultivation, approximately 5 × 10^8^ cells were collected and centrifuged at 5,000 × g for 10 min. The cell pellets were immediately resuspended in 500 μL of RNA solution (Ambion, UK) and RNA was extracted using a total RNA isolation kit (Macherey-Nagel, Germany). One microgram of total RNA was employed to synthesize the first-strand cDNA in a 20 µL reaction using the SuperScript III first-strand synthesis system (Invitrogen, USA). A real-time PCR analysis was performed, according to the SYBR green method, in a 20 µL reaction volume using the StepOne Real Time PCR system (Applied Biosystems, USA). The PCR efficiencies of all of the primers were experimentally determined and found to be suitable for reliable copy-number quantification. The mRNA quantity was estimated based on ΔΔCT value, as described previously^25^. The assays were performed in duplicate, and a template-less reaction was used as a negative control.

### Construction plasmids

Supplementary Table S1 lists the bacterial strains and plasmids used in this study. *E*. *coli* BL21 (DE3) served as the host for protein production, and *E*. *coli* Top10 was used for routine cloning and plasmid maintenance. The LB medium was used for the growth of *E*. *coli*. Ampicillin at 100 mg/L was added to the culture medium when needed. Gene manipulation was carried out using standard methods^7^. The plasmid pET30b(+) was used to clone the *mmsR* gene in *E*. *coli* BL21(DE3). The *mmsR* gene was amplified from *P*. *denitrificans* genomic DNA by PCR and the fragment was ligated into the pET30b(+) vector using a seamless cloning and assembly kit (Invitrogen). The resulting plasmid, pET30b(+)/*mmsR*, which contained the ET-*mmsR* gene with the His-tag at the *C*-terminus, was sequenced by Macrogen Co., Ltd., Korea, and introduced into *E*. *coli* BL21(DE3).

The plasmid pUCPK was used to develop3-HP biosensor in *P*. *denitrificans*. The sequences for *UC*-*Pc1*, *UC*-*mmsR* and *UC*-*P*_*mmsA*_ were amplified from *P*. *denitrificans* genomic DNA by PCR, while the *UC*-*gfp* gene, encoding the green fluorescence protein (GFP), was amplified from pPro24-*gfp* (Addgene). Four DNA fragments of *UC*-*Pc1*, *UC*-*mmsR*, *UC*-*P*_*mmsA*_ and *UC*-*gfp* were overlapped and ligated into the pET30b(+) vector using a seamless cloning and assembly kit (Invitrogen). The resulting plasmid, pUCPK-P_*c1*_-*mmsR*-P_*mmsA*_-*gfp*, was sequenced by Macrogen Co., Ltd., Korea, and introduced into *P*. *denitrificans*. Details of primers are given in Supplementary Table S3.

Plasmids used in deletion analysis and site-directed mutagenesis were constructed by amplifying various target fragments, such as *P*_*wt*-*gfp*, *P*_*Δ1*-*gfp*, *P*_*Δ*2-*gfp*, *P*_*Δ3*-*gfp*, *P*_*Δ4*-*gfp*, *P*-1*0*^*mut*^-*gfp*, *P*-*35* ^*mut*^-*gfp*, *O*_*wt*-*gfp*, *O*_ *Δ1*-*gfp*, *O*_ *Δ*2-*gfp*, *O*_ *Δ3*-*gfp*, *O_ Δ4*-*gfp*, *O*_*1*_^*mut*^-*gfp*, *O*_*2*_^*mut*^-*gfp and O*_*1*_^*mut*^*O*_*2*_^*mut*^-*gfp*, using the recombinant pUCPK-P_*c1*_-*mmsR*-P_*mmsA*_-*gfp* plasmid as template with the primers shown in Supplementary Table S3. The amplified PCR fragments were ligated into the pUCPK or pUCPK-P_*c1*_-*mmsR* vectors (Supplementary Table S3) using a seamless cloning and assembly kit (Invitrogen). The resulting plasmids was sequenced by Macrogen Co., Ltd., Korea, and introduced into *P*. *denitrificans*WT or *P*. *denitrificans*Δ*mmsR*.

### Transcription start site identification

To determine the 5′ end of the *mmsA* transcript, 5′ rapid amplification of cDNA ends (RACE) PCR was performed using a SMARTer® RACE 5′/3′ kit (Clontech, USA). The RACE experiment was carried out according to the manufacturer’s instruction. Synthesis of the first strand cDNA was performed on 1 μg of the DNase-treated total RNA of *P*. *denitrificans* (wild-type) grown under induction condition as described before. RACE fragments were purified from agarose gels using the Qiagen gel-extraction kit and cloned into the linearized pRACE vector with In-Fusion HD Cloning (Takara, Japan). DNA from seven colonies of each RACE synthesis was sequenced using M13 forward and reverse primers. Primer extension assays were performed as previously described (Zeng *et al*. 2002), except that 50 µg of total RNA, derived from transfected 293 T cells, was used. The sequence of gene-specific primer (*mmsA*-GSP5-RP) used for RACE was: ′5-GATTACGCCAAGCTTCGGCCCGAGGTCGCCCAGCTTGGAGCCGCG-3′.

### Expression and purification of MmsR protein

For the production of soluble His-tagged MmsR, pET30b(+)/*mmsR* was co-expressed with various chaperone plasmids, i.e. pG-KJE8, pGRO7, pKJE7, pG-TF2, and pTF-16 (Takara, Japan), in *E*. *coli* BL21(DE3). The recombinant BL21(DE3) strains were grown in LB medium supplemented with 50 mg/L kanamycin, 25 mg/L chloramphenicol and/or 0.5 mg/mL L-arabinose. The cells were grown aerobically in 1 L Erlenmeyer flasks containing 350 mL of medium at 30 °C and 200 rpm in an orbital incubator shaker. The cells were induced at ~0.6 OD_600_ with 0.1 mM isopropyl-beta-D-thiogalactopyranoside (IPTG) and grown at 25 °C, 150 rpm for 10 h. The cells were then harvested and centrifuged at 10,000 × g for 10 min. The pellets were washed with 100 mM potassium phosphate buffer (pH 7.0) and resuspended in the binding buffer (20 mM sodium phosphate buffer containing 0.5 M NaCl and 20 mM imidazole). The resuspended cells were disrupted using the French Pressure Cell (FA-078A, Thermo Electron Corp.; Waltham, MA, USA) at 1,250 psi. The cell lysate was centrifuged at 15,000 × g at 4 °C for 30 min to remove the particulate fraction and unbroken cells. The soluble fraction was subjected to purification under non-denaturing conditions by Ni-affinity chromatography using a Ni-NTA-HP resin column (17-5248-01; GE Healthcare, Sweden). The eluents from the column were pooled and dialyzed using a 10 kDa cut-off membrane to remove the salts. The resulting enzyme extract was electrophoresed under denaturing and non-denaturing conditions, as described by Laemmli, and the extract was stored at −80 °C in 20% glycerol.

### DNase I footprinting analysis of the mmsA promoter

DNase I footprinting analysis was conducted using an automated capillary sequencer^26^. FAM-labeled fragments of the *mmsA* promoter were synthesized by a standard PCR method using a FAM-labeled oligonucleotide (Macrogen, Korea) and *P*. *denitrificans* genomic DNA as template, purified on agarose gel and extracted from the gel (Qiagen). The 250 ng DNA fragment (169 bp) was incubated with the recombinant MmsR (0.15, 0.6 and 1.2 µM) for protection at room temperature for 40 sec in 10 µl 1X binding buffer which contains 50 mM Tris (pH 7.4), 750 mM KCl, 0.5 mM dithiothreitol and 0.5 mM EDTA. Protected DNA fragments were digested with 0.05 units of DNase I at room temperature for 30 sec in the presence of 1X DNase I buffer, and the digestion reaction was stopped by adding the stop solution containing EDTA (pH 8.0) to a final concentration of 250 mM. Afterwards, the sample was heated to 70 °C and rapidly cooled to 4 °C. Denatured DNA fragments were separated on an ABI 3130xl Genetic analyzer (36 cm capillary array and POP4 polymer; Applied Biosystems Inc., Foster City, CA, USA). A 1.5 µL of assay product was mixed with 8.3 µL of deionized formamide (Applied Biosystems) and 0.2 µL of ROX 500 size-standard (Applied Biosystems Inc.), the latter of which wasdenatured at 95 °C for 5 min and quenched at 4 °C for 3 min. The mixture was injected into capillaries by applying 1.6 KV for 15 sec, and then electrophoresed under 10 kV and an operating temperature of 60 °C. Data analysis was conducted by GeneMapper software (version 4.1, Applied Biosystems). DNA fragments labeled with ROX were used as internal standard (Invitrogen, USA).

### Electrophoretic Mobility Shift Assay (EMSA)

The 130 bp-long DNA fragments, designated F_12_, F_12M_, F_1M2_ and F_1M2M_, were PCR-amplified (Supplementary Table S2) and purified using a glass fiber column (PCR purification kit, Qiagen). EMSA was performed using Molecular Probes’ Fluorescence-based Electrophoretic Mobility Shift Assay (fluorescence-EMSA) kit (Invitrogen, E33075), following the standard protocols supplied by the manufacturer. Binding reactions were assembled on ice with increasing amounts of purified MmsR protein (34.4 kDa) added separately to a fixed concentration (40 nM) of the DNA fragments (F_12_, F_12M_, F_1M2_ and F_1M2M_) in a final 12 µl volume of 1X binding buffer (50 mM Tris (pH 7.4), 750 mM KCl, 0.5 mM dithiothreitol, 0.5 mM EDTA). The reaction was incubated at room temperature for 30 min before loading onto 6% non-denaturating polyacrylamide gel. The gel was run at 200 V in pre-chilled 0.5X TBE buffer (44.5 mM Tris base, 44.5 mM boric acid, 0.5 mM EDTA; pH~8) for 30 min. The gel was fixed and stained with SYBR Green EMSA stain for DNA observation or SYPRO Ruby EMSA stain for protein observation, before quantification on a gel documentation system (Bio-Rad systems).

### Statistical analysis

Values form RNA transcription level or fluorescence (AU/OD_600_) were confirmed by one-way analysis of variance (ANOVA) with tukey post hoc comparisons for each dependent variable at 95% of confidence interval.

## Supplementary information

Supplementary Information
