# Supplementary material for: A novel 3-hydroxypropionic acid-inducible promoter regulated by the LysR-type transcriptional activator protein MmsR of Pseudomonas denitrificans
Source: Sci Rep. 2019 Mar 29;9:5333. doi: 10.1038/s41598-019-41785-y (PMC6441110; doi:10.1038/s41598-019-41785-y)
Supplement: Supplementary file 1 — Supplementary Information [file 41598_2019_41785_MOESM1_ESM.pdf]

A novel 3-hydroxypropionic acid-inducible promoter regulated by the LysR-type transcriptional activator protein MmsR of *Pseudomonas denitrificans*

Nam Hoai Nguyen[a], Satish Kumar Ainala[b], Shengfang Zhou[c], Sunghoon Park[a,b]*

[a]School of Chemical and Biomolecular Engineering, Pusan National University, Busan 609-735, Republic of Korea

[b]School of Energy and Chemical Engineering, UNIST, UNIST-gil 50, Ulsan 689-798, Republic of Korea

[c]The Key Laboratory of Biotechnology for Medicinal Plant of Jiangsu Province, Jiangsu Normal University, No. 101 Shanghai Road, Tongshan District, Xuzhou City, Jiangsu Province, 221116, China

***Corresponding author**

Affiliation: School of Energy and Chemical Engineering, UNIST

Mailing Address: School of Energy and Chemical Engineering, UNIST, UNIST-gil 50, Ulsan 689-798, Republic of Korea

Phone: +82-52-217-2565

Fax: +82-52-217-2309

E-mail: parksh@unist.ac.kr

Supplementary Figures and Tables

Supplementary Fig. S1. Identification of the *mmsA* transcription start site (TSS). TSS was determined by the method called 5' rapid amplification of cDNA ends (RACE). The nucleotide 'G' in the black box indicates the 5' end which is the TSS of *mmsA*. The detail of primer and template sequences was showed the figure.

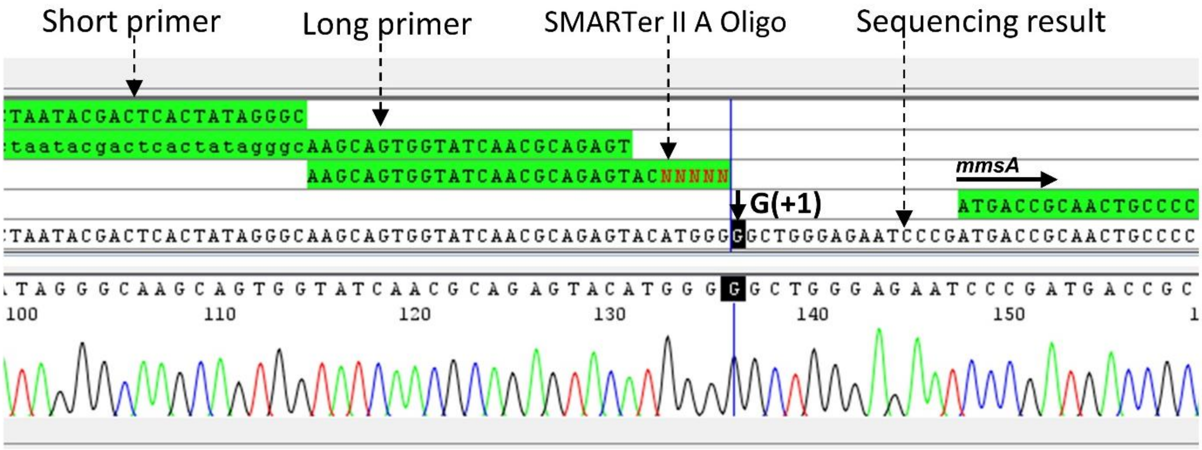

Supplementary Fig. S2. **(a)** Secondary structure formation of the *mmsR* and *mmsA* intergenic region (136 bp) using mfold web server. Putative O_1 was suggested due to the best steem-loop structure. The 5'- and 3'-ends indicated the start codons of *mmsR* and *mmsA*, respectively. The minimum free energy, ΔG, was -13.01 kcal/mol. **(b)** Identification of O_2 site by using palindromic sequence in O_1 as query sequence. The letter showed in red indicates the repeat inverted region which form hairpin structure. Promoter elements (-10, -35) and TSS (+1) of P_{mmsA} promoter were underlined. The red x symbols indicate the mismatch between candidate and query sequence. **(c)** Comparing locations of activator binding sites and promoter regions in other LTTR system. Black bars and red numbers indicate the promoter regions. Black arrows indicated the transcription start sites. Gray and white boxes indicate the half-sites and spacers of operator regions.

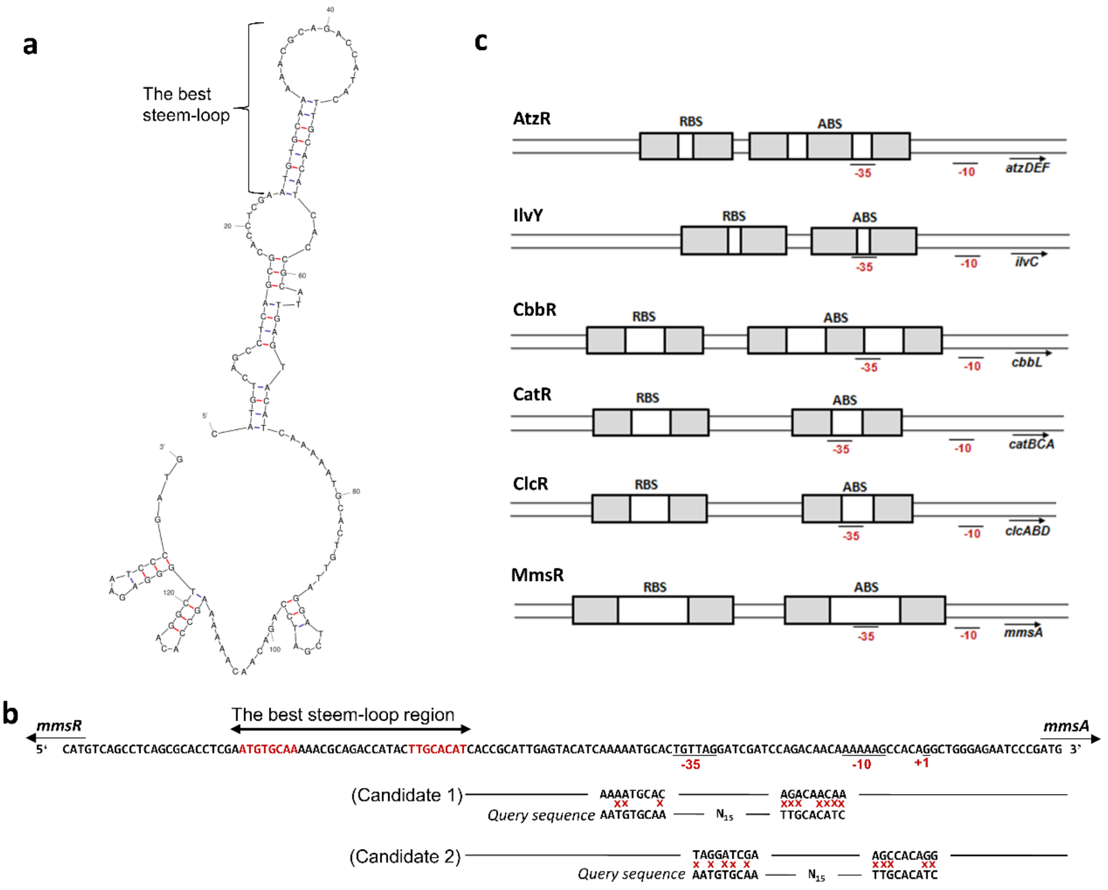

Supplementary Fig. S3. Relative mRNA expression levels of the *mmsA* gene in *P. denitrificans*WT or *P. denitrificans*Δ*mmsR* containing recombinant *mmsR* gene in N-terminal His-tagged (N-his-*mmsR*-C) or C-terminal His-tagged (C-his-*mmsR*-C) form. 3-HP was not (black bar) or supplemented at 25 mM (red bar). The data were normalized against the un-induced mRNA expression level of *mmsA* in the wildtype strain, which was ~10-fold lower than that of the reference gene, *rpoD*.

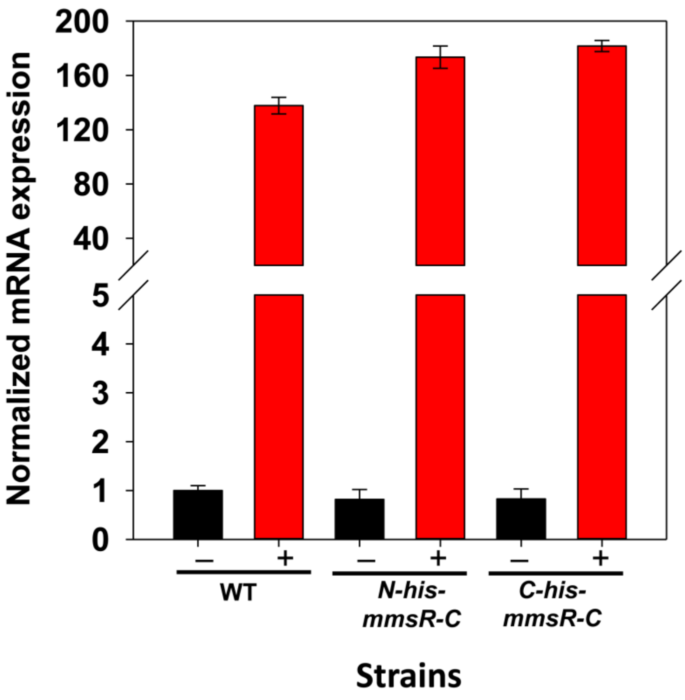

Supplementary Fig. S4. SDS-PAGE analyses to study the solubility of recombinant C-His-tagged MmsR protein with the help of various chaperon plasmids such as pG-KJE8 **(a)**, pGro7 **(b)**, pKJE7 **(c)**, pG-Tf2 **(d)**, and pTf16 **(e)** in recombinant *E. coli* BL21. Total (T) and soluble (S) fractions of crude cell-extracts were analyzed. Cells were cultured in the absence (–) or presence (+) of 25 mM 3-HP in LB medium at 25 °C and 150 rpm, and harvested at 4 h and 12 h after induction with 0.1 mM IPTG. The blue arrows indicate MmsR protein at 34.4 kDa. Crude cell-extract of *E. coli* BL21 host ('C') and protein markers ('M') (Fermentas #SM1811) are also shown.

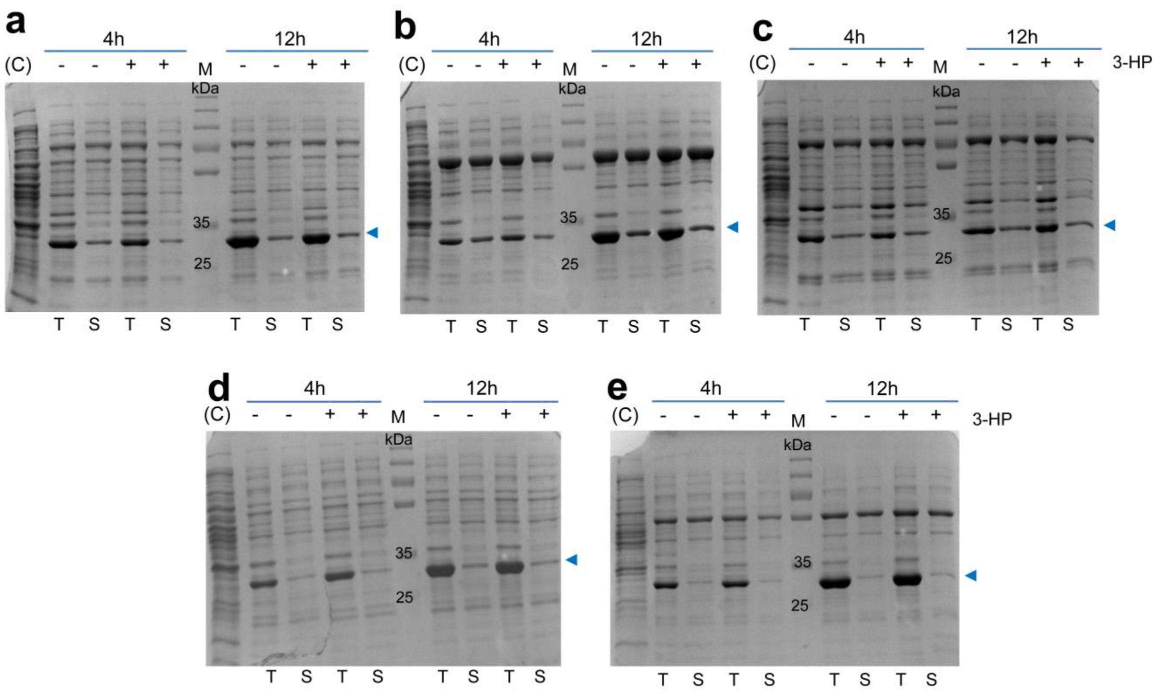

Supplementary Fig. S5. **(a)** SDS-PAGE showing expression and purification of recombinant MmsR protein. Lane 1, host *E. coli* BL21 (cell-free extract); lane 2, recombinant *E. coli* BL21 co-expressing pKJE7 and pUCPK'/P$_{c1}$_*mmsR*-*C6xhis* cultured IPTG induction (cell-free extract); lanes 4 and 5, correspond to binding steps; lane 6 and 7 correspond to washing steps; lane 8, 9 and 10 correspond to elution steps from recombinant *E. coli* BL21. Protein markers (Fermentas #SM1811) are shown in lanes 3. The blue indicates the purified MmsR protein. **(b)** Blue Native-PAGE showing oligomeric forms of recombinant MmsR protein. Lanes 2, 4, and 6 correspond to the purified MmsR protein at 65, 220, 550 nM, respectively. Protein markers (Fermentas #SM1811) are shown in lanes 1, 3, and 5. The red arrows indicate the purified MmsR protein, in different oligomeric forms. Fig. S4b was cropped from full-length gels shown in Supplementary Fig. S6.

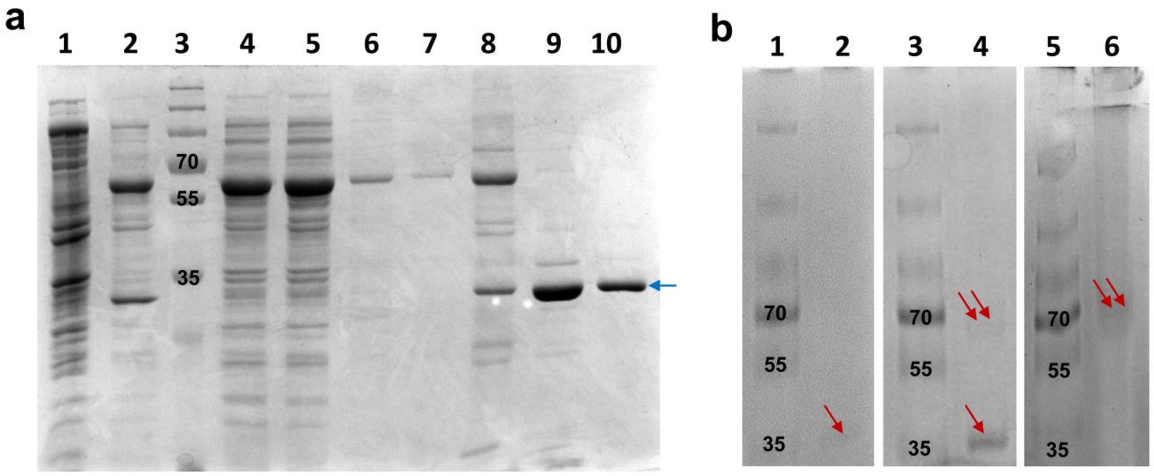

Supplementary Fig. S6. Full-length gels for *in vitro* electromobility shift assay (EMSA) to study binding of the MmsR protein to F_{12M} (a) or F_{1M2} (b) in the absence (-) or presence (+) of 25 mM 3-HP. Lanes 1 or 6, 2 or 7, 3 or 8, 4 or 9 and 5 or 10 correspond to 0, 0.73, 2.9, 11.6 and 48.5 nM of the MmsR protein concentration, respectively. The concentration of DNA fragments was fixed at 40 nM, excepting lane a2 (empty DNA as a control).

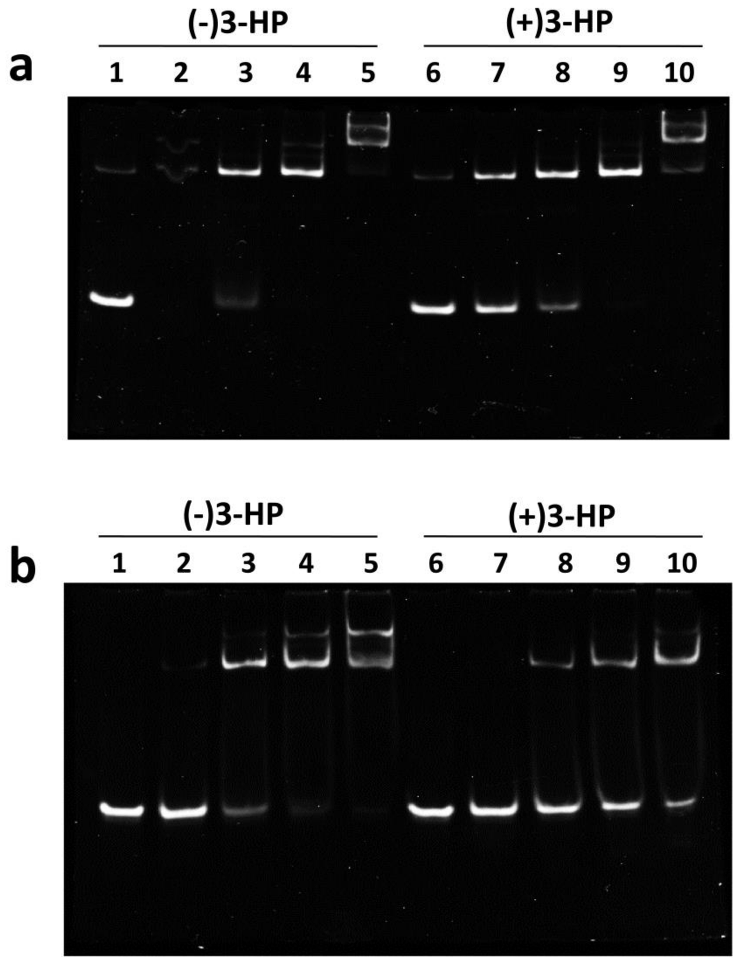

Supplementary Fig. S7. Full-length gels for Blue Native-PAGE experiment. Lanes 1 and 3 correspond to the purified MmsR protein at 65 nM; lanes 4 and 6 correspond to the purified MmsR protein at 130nM; lanes 7 and 9 correspond to the purified MmsR protein at 220; lanes 13, 15, 17 and 20 correspond to the purified MmsR protein at 400, 450, 500 and 550 nM, respectively. Protein markers (Fermentas #SM1811) are shown in lanes 2, 5, 8, 11 and 19. The other lanes 10, 12, 14, 16 and 18 were empty.

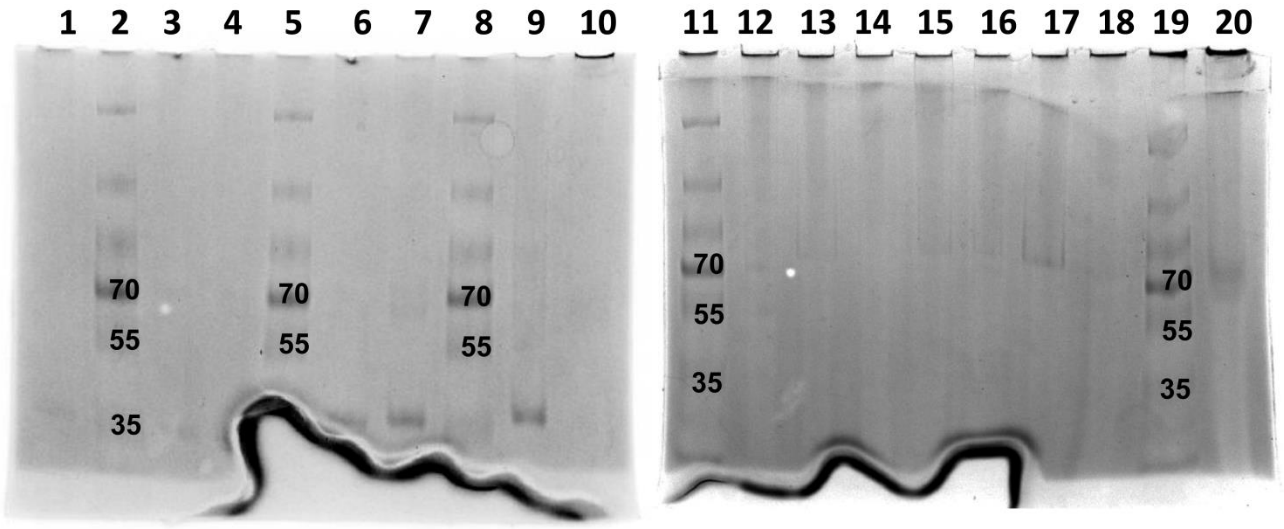

Supplementary Table S1. Bacterial strains, plasmids and used in this study.

Strains and Plasmids	Description	Source
**Strains**		
E. coli		
Top10	Cloning host	Invitrogen
BL21 (DE3)	Expression host	
P. denitrificans		
WT	*P. denitrificans* ATCC13867; Source for *mmsR* and *mmsA* genes	KCCM, Korea
ΔmmsR	*P. denitrificans* ATCC13867Δ*mmsR* single mutant strain	This study
ΔΔΔ	*P. denitrificans* ATCC13867Δ*3hpdh*Δ*3hibdhIV*Δ*3hibdhI*	Previous study
mmsR-C	*P. denitrificans* ATCC13867Δ*mmsR* harboring pUCPK'/P_{C1}_*mmsR*	This study
**Plasmids**		
pQSAK	*sacB-Km* cassette cloned into pQE-80L; Km^r& Amp^r	Zhou et al. 2014
pQSAK/*ΔmmsR*	Plasmid used to delete *mmsR* gene in *P. denitrificans* ATCC13867 wt	This study
pUCPK'	Modified pUCPK plasmid with reduced size; Km^r	Previous study
pUCPK'/P_{c1}_*mmsR*	*mmsR* gene in pUCPK' plasmid; Km^r	This study
pUCPK'/P_{c1}_*mmsR-N6xhis*	*mmsR* gene and N-*his-tag* in pUCPK' plasmid; Km^r	This study
pUCPK'/P_{c1}_*mmsR-C6xhis*	*mmsR* gene and C-*his-tag* in pUCPK' plasmid; Km^r	This study
pET30b/*mmsR*	*mmsR* gene in pET30b plasmid; Amp^r	This study
pG-KJE8	*dnaK, dnaJ, grpE, groES, groEL* chaperone genes in plasmid; Cm^r	Takara, Japan
pGro7	*groES, groEL* chaperone genes in plasmid; Cm^r	Takara, Japan
pKJE7	*dnaK, dnaJ, grpE* chaperone genes in plasmid; Cm^r	Takara, Japan
pG-Tf2	*groES, groEL, tig* chaperone genes in plasmid; Cm^r	Takara, Japan
pTf16	*tig* chaperone genes in plasmid; Cm^r	Takara, Japan
pUCPK-P_{c1}-*mmsR*-P_{mmsA}-*gfp*	Biosensor; Km^r	This study
pUCPK-P_{c1}-*mmsR*-P_{mmsR}-*gfp*	*gfp* under the control of mmsR native promoter, P_{mmsR}; Km^r	This study
P_wt	Plasmid containing sequence from -115 to + 19; Km^r	This study
P_Δ1	Plasmid containing sequence from -58 to + 19; Km^r	This study
P_Δ2	Plasmid containing sequence from -35 to + 19; Km^r	This study
P_Δ3	Plasmid containing sequence from -24 to + 19; Km^r	This study
P_Δ4	Plasmid containing sequence from -14 to + 19; Km^r	This study
P-10^{mut}	Plasmid containing mutation of -10 region; Km^r	This study
P-35^{mut}	Plasmid containing mutation of -35 region; Km^r	This study
O_wt	Plasmid containing *mmsR* and sequence from -115 to + 19; Km^r	This study
O_Δ1	Plasmid containing *mmsR* and sequence from -98 to + 19; Km^r	This study
O_Δ2	Plasmid containing *mmsR* and sequence from -79 to + 19; Km^r	This study
O_Δ3	Plasmid containing *mmsR* and sequence from -48 to + 19; Km^r	This study
O_Δ4	Plasmid containing *mmsR* and sequence from -28 to + 19; Km^r	This study
O_1^{mut}	Plasmid containing *mmsR* and mutation of O_1 region; Km^r	This study
O_2^{mut}	Plasmid containing *mmsR* and mutation of O_2 region; Km^r	This study
$O_1^{mut}O_2^{mut}$	Plasmid containing *mmsR* and mutation of O_1 & O_2; Km^r	This study

Supplementary Table S2. Fragments for EMSA used in this study.

Fragments	Sequence (5' – 3')[a]	Source
F_{12}	GTCAGCCTCAGCGCACCTCG**AATGTGCAA**AAACGCAGACCATAC**TTGCACATC**ACCGCATTGAGTACATCA*AAAATGCAC*TGTTAGGATCGATCC*AGACAACAA*AAAAGCCACAGGCTGGGAGAATCCCG	This study
F_{12M}	GTCAGCCTCAGCGCACCTCG**AATGTGCAA**AAACGCAGACCATAC**TTGCACATC**ACCGCATTGAGTACATCA*ATCCTTGCT*TGTTAGGATCGCGCC*CAGGCACAA*AAAAGCCACAGGCTGGGAGAATCCCG	This study
F_{1M2}	GTCAGCCTCAGCGCACCTCG**ATCCTTGCT**AAACGCAGACCATAC**CGCCCAGGC**ACCGCATTGAGTACATCA*AAAATGCAC*TGTTAGGATCGATCC*AGACAACAA*AAAAGCCACAGGCTGGGAGAATCCCG	This study
F_{1M2M}	GTCAGCCTCAGCGCACCTCG**ATCCTTGCT**AAACGCAGACCATAC**CGCCCAGGC**ACCGCATTGAGTACATCA*ATCCTTGCT*TGTTAGGATCGCGCC*CAGGCACAA*AAAAGCCACAGGCTGGGAGAATCCCG	This study
DNase I footprinting fragment	CCGCAGGTTGTCCCAGTCCATGTCAGCCTCAGCGCACCTCG**AATGTGCAA**AAACGCAGACCATAC**TTGCACATC**ACCGCATTGAGTACATCA*AAAATGCAC*TGTTAGGATCGATCC*AGACAACAA*AAAAGCCACAGGCTGGGAGAATCCCGATGACCGCAACTGCCCCG	This study

[a]Bold letter indicated the half sites of O_1 operator; italic letter indicated the half sites of O_2 operator; underlined letter indicated site-directed mutagenesis region.

Table S3. Cloning information

PCR fragment	Primer	Primer sequence (5'- 3')	Template	Plasmid backbone
QS-*mmsR*-US	F1<br>F2	CCCTTTCGTCTTCACCTCGAGGGAACATCCAC<br>GCGCCCTACCAGTCCATGTCAGC	gDNA P. denitrificans	pQSAK / XbaI / XhoI
QS-*mmsR*-DS	F3<br>F4	TGGACTGGTAGGGCGCATAAATCG<br>AAAATGCCGATTCTAGAAGCGCGTGGTCTAC	gDNA P. denitrificans	
UC-Pc1	F5<br>F6	TTGTAAAACGACGGCCAGTGAATTCAACTGCCGCAAGCCCAGTT<br>TGCCGGCGCGGGAAAGCTCGAGGAAAAACCGCAGGTTGTCCCAGTCCATGCTGGTTTCCTCCTGT	gDNA P. denitrificans	pUCPK<br>/ EcoRI / HindIII
UC-*mmsR*	F7<br>F8	GGCTGTCGCGGCGAGGGACTACATTCCCCGCCCCATCAA<br>GAACAGGAGGAAACCAGCATGGACTGGGACAACCTG	gDNA P. denitrificans	
UC-mmsR-C6xhis	F5<br>F9	TTGTAAAACGACGGCCAGTGAATTCAACTGCCGCAAGCCCAGTT<br>CAGGTCGACTCTAGAGGATCCAAGATCACCTTGCGCTCTT	pUCPK-Pc1-mmsR	pUCPK<br>/ EcoRI / HindIII
UC-*mmsR*-N6xhis	F5<br>F10	TTGTAAAACGACGGCCAGTGAATTCAACTGCCGCAAGCCCAGTT<br>TTGCCGGCGCGGGAAAGCTCGAGGAAAAA	pUCPK-Pc1-mmsR	pUCPK<br>/ EcoRI / HindIII
ET-*mmsR*	F11<br>F12	TGGTGGTGGTGGTGCTCGAGCATTCCCCGCCCCATCAA<br>AAGAAGGAGATATACATATGATGGACTGGGACAACCTGCGG	gDNA P. denitrificans	pET30b+ / XhoI / NdeI
UC-P_{mmsA}	F13<br>F14	CCGATTTATGCGCCTCTAGAGGATCCCCGGGTACTAACATCATGCTGACCTCAGCGCACCTC<br>GATTTATGCGCCTCTAGAGGATCCCCGGGTACTAACATGTCAGCCTCAGCGCA	gDNA P. denitrificans	pUCPK-P_{c1}-*mmsR*<br>/BsrGI / HindIII
UC-*gfp*	F15<br>F16	GGCTGTCGCGGCGAGGGATTAATGGTGATGGTGATGGTG<br>GCCACAGGCTGGGAGAATCCCGATGCAGAGCAAAGGCGAAG	pPro24-*gfp*	
P_wt-*gfp*	F13<br>F16	CCGATTTATGCGCCTCTAGAGGATCCCCGGGTACTAACATCATGCTGACCTCAGCGCACCTC<br>GCCACAGGCTGGGAGAATCCCGATGCAGAGCAAAGGCGAAG	pUCPK-P_{c1}-*mmsR*-P_{mmsA}-*gfp*	pUCPK<br>/ EcoRI / HindIII
P_Δ1-*gfp*	F17<br>F16	CCGATTTATGCGCCTCTAGAGGATCCCCGGGTACTAACATGACCATACTTGCACATC<br>GCCACAGGCTGGGAGAATCCCGATGCAGAGCAAAGGCGAAG	pUCPK-P_{c1}-*mmsR*-P_{mmsA}-*gfp*	pUCPK<br>/ EcoRI / HindIII
P_Δ2-*gfp*	F18<br>F16	CCGATTTATGCGCCTCTAGAGGATCCCCGGGTACTAACATTGTTAGGATCGATCCAGAC<br>GCCACAGGCTGGGAGAATCCCGATGCAGAGCAAAGGCGAAG	pUCPK-P_{c1}-*mmsR*-P_{mmsA}-*gfp*	pUCPK<br>/ EcoRI / HindIII
P_Δ3-*gfp*	F19<br>F16	CCGATTTATGCGCCTCTAGAGGATCCCCGGGTACTAACATATCCAGACAACAAAAAAGCC<br>GCCACAGGCTGGGAGAATCCCGATGCAGAGCAAAGGCGAAG	pUCPK-P_{c1}-*mmsR*-P_{mmsA}-*gfp*	pUCPK<br>/ EcoRI / HindIII
P_Δ4-*gfp*	F20<br>F16	CCGATTTATGCGCCTCTAGAGGATCCCCGGGTACTAACATCAAAAAAGCCACAGGCTGGG<br>GCCACAGGCTGGGAGAATCCCGATGCAGAGCAAAGGCGAAG	pUCPK-P_{c1}-*mmsR*-P_{mmsA}-*gfp*	pUCPK<br>/ EcoRI / HindIII
O_wt-*gfp*	F21<br>F16	GGTATTGCAGCTCCTTGCTGTACAACAGGTCATCGACG<br>GCCACAGGCTGGGAGAATCCCGATGCAGAGCAAAGGCGAAG	pUCPK-P_{c1}-*mmsR*-P_{mmsA}-*gfp*	pUCPK-P_{c1}-*mmsR*<br>/BsrGI / HindIII
O_Δ1-*gfp*	F22<br>F16	GGTATTGCAGCTCCTTGCTGTACAATCGAATGTGCAAAAACGC<br>GCCACAGGCTGGGAGAATCCCGATGCAGAGCAAAGGCGAAG	pUCPK-P_{c1}-*mmsR*-P_{mmsA}-*gfp*	pUCPK-P_{c1}-*mmsR*<br>/BsrGI / HindIII
O_Δ2-*gfp*	F23<br>F16	GGTATTGCAGCTCCTTGCTGTACAAGACCATACTTGCACATC<br>GCCACAGGCTGGGAGAATCCCGATGCAGAGCAAAGGCGAAG	pUCPK-P_{c1}-*mmsR*-P_{mmsA}-*gfp*	pUCPK-P_{c1}-*mmsR*<br>/BsrGI / HindIII
O_Δ3-*gfp*	F24<br>F16	GGTATTGCAGCTCCTTGCTGTACAAATCAAAAATGCACTGTTAGG<br>GCCACAGGCTGGGAGAATCCCGATGCAGAGCAAAGGCGAAG	pUCPK-P_{c1}-*mmsR*-P_{mmsA}-*gfp*	pUCPK-P_{c1}-*mmsR*<br>/BsrGI / HindIII
O_Δ4-*gfp*	F25<br>F16	GGTATTGCAGCTCCTTGCTGTACAACAGACAACAAAAAAGCCACAGG<br>GCCACAGGCTGGGAGAATCCCGATGCAGAGCAAAGGCGAAG	pUCPK-P_{c1}-*mmsR*-P_{mmsA}-*gfp*	pUCPK-P_{c1}-*mmsR*<br>/BsrGI / HindIII

P-10^{mut}	F13	CCGATTTATGCGCCTCTAGAGGATCCCCGGGTACTAACATCATGCTGACCTCAGCGCACCTC	pUCPK-P_{c1}-*mmsR*-P_{mmsA}-*gfp*	pUCPK / EcoRI / HindIII
	F27	CCAGCCTGTGGTCGCACTGTTGTCTGGAT		
-10^{mut}-*gfp*	F28	ATCCAGACAACAGTGCGACCACAGGCTGG	pUCPK-P_{c1}-*mmsR*-P_{mmsA}-*gfp*	
	F16	GCCACAGGCTGGGAGAATCCCGATGCAGAGCAAAGGCGAAG		
P-35^{mut}	F13	CCGATTTATGCGCCTCTAGAGGATCCCCGGGTACTAACATCATGCTGACCTCAGCGCACCTC	pUCPK-P_{c1}-*mmsR*-P_{mmsA}-*gfp*	pUCPK / EcoRI / HindIII
	F29	GTCTGGATCGATCGGCTGCGTGCATTTTT		
-35^{mut}-*gfp*	F30	AAAAATGCACGCAGCCGATCGATCCAGAC	pUCPK-P_{c1}-*mmsR*-P_{mmsA}-*gfp*	
	F16	GCCACAGGCTGGGAGAATCCCGATGCAGAGCAAAGGCGAAG		
O_1^{mu}t	F21	GGTATTGCAGCTCCTTGCTGTACAACAGGTCATCGACG	F1M2 fragment	pUCPK-P_{c1}-*mmsR* /BsrGI / HindIII
	F14	GATTTATGCGCCTCTAGAGGATCCCCGGGTACTAACATGTCAGCCTCAGCGCA		
O_1^{mut}-*gfp*	F15	GGCTGTCGCGGCGAGGGATTAATGGTGATGGTGATGGTG	pUCPK-P_{c1}-*mmsR*-P_{mmsA}-*gfp*	
	F16	GCCACAGGCTGGGAGAATCCCGATGCAGAGCAAAGGCGAAG		
O_2^{mut}	F21	GGTATTGCAGCTCCTTGCTGTACAACAGGTCATCGACG	F12M fragment	pUCPK-P_{c1}-*mmsR* /BsrGI / HindIII
	F14	GATTTATGCGCCTCTAGAGGATCCCCGGGTACTAACATGTCAGCCTCAGCGCA		
O_2^{mut}-*gfp*	F15	GGCTGTCGCGGCGAGGGATTAATGGTGATGGTGATGGTG	F1M2 fragment	
	F16	GCCACAGGCTGGGAGAATCCCGATGCAGAGCAAAGGCGAAG		
$O_1^{mut}O_2^{mut}$	F21	GGTATTGCAGCTCCTTGCTGTACAACAGGTCATCGACG	F1M2M fragment	pUCPK-P_{c1}-*mmsR* /BsrGI / HindIII
	F14	GATTTATGCGCCTCTAGAGGATCCCCGGGTACTAACATGTCAGCCTCAGCGCA		
$O_1^{mut}O_2^{mut}$-*gfp*	F15	GGCTGTCGCGGCGAGGGATTAATGGTGATGGTGATGGTG	pUCPK-P_{c1}-*mmsR*-P_{mmsA}-*gfp*	
	F16	GCCACAGGCTGGGAGAATCCCGATGCAGAGCAAAGGCGAAG		
